# Cost-Effective Fabrication of Modified Palygorskite-Reinforced Rigid Polyurethane Foam Nanocomposites

**DOI:** 10.3390/nano12040609

**Published:** 2022-02-11

**Authors:** Yulei Wang, Kaibin Cui, Baizeng Fang, Fei Wang

**Affiliations:** 1 Key Laboratory of Special Functional Materials for Ecological Environment and Information, Hebei University of Technology, Ministry of Education, Tianjin 300130, China; wangyuulei@126.com (Y.W.); cuikaibin2021@163.com (K.C.); 2 Department of Chemical & Biological Engineering, University of British Columbia, 2360 East Mall, Vancouver, BC V6T 1Z3, Canada

**Keywords:** rigid polyurethane foam nanocomposites, modified palygorskite, cellular structure, mechanical properties, thermal stability, insulation nanomaterials

## Abstract

Integration of nanoclay minerals into rigid polyurethane foams (RPUFs) is a cost-effective solution to enhance foam’s performance via environmental protection technology. In this work, palygorskite/RPUFs nanocomposites (Pal/RPUFNs) with excellent mechanical properties and thermal stability were prepared via a one-step method, using 4,4’-diphenylmethane diisocyanate and polyether polyol as the starting materials, coupled with Pal modified by silane coupling agent KH570. The effects of the modified Pal on the mechanics, morphology, and thermal properties of the nanocomposites were studied systematically. When the content of the modified Pal was 8 wt% of polyether polyol, the elastic modulus and compressive strength of the Pal/RPUFNs were increased by ca. 131% and 97%, respectively. The scanning electron microscopy images indicated that the addition of the modified Pal significantly decreased the cell diameter of the Pal/RPUFNs. The results of thermogravimetric and derivative thermogravimetry analyses revealed that the addition of the modified Pal increased the thermal weight loss central temperature of the Pal/RPUFNs, showing better thermal stability in comparison with the pure RPUFs. A self-made evaluation device was used to estimate the thermal insulation ability of the Pal/RPUFNs. It was found that the small cell size and uniform cellular structure were keys to improving the thermal insulation performance of the RPUFs. The prepared Pal/RPUFNs are expected to have great potential in the field of building insulation.

## 1. Introduction

Polyurethane is a special polymer material that has been widely used in the construction, automobile, aerospace, light, and chemical industries owing to its unique physical and chemical properties [1,2,3]. Due to their low density, low thermal conductivity, and high commercial application value, rigid polyurethane foams (RPUFs) have been considered to be the best insulation materials among various types of polyurethanes [4]. One of the most common synthesis methods for RPUFs is the one-step method, based on the main chain urethane group formation involving the reaction of polyol and isocyanate [5]. In the above process, the blowing agents are often used to produce cells within the foam. Additional regulators containing catalysts and surfactants are also added to adjust the physical and chemical properties of RPUFs [6]. With the increase in people’s interest in RPUFs and their wide range of applications, improving the comprehensive performance of RPUFs, particularly mechanical property and thermal stability, has become an important topic [7]. The addition of fillers has been regarded as an effective route to the reinforcement of RPUFs. In recent years, different types of fillers have been incorporated into RPUFs, such as carbon fibers, carbon nanotubes, potato protein, lignin, tire rubber, etc. [2,8,9,10,11]. Although the above fillers are efficient in enhancing the mechanical properties of RPUFs, some of them can cause harmful effects on live organisms. As we know, carbon fibers and carbon nanotubes produce toxic reactions when they reach the lungs in a large quantity and have high persistence in the environment, which causes health problems to live organisms [12]. Moreover, carbon fibers and carbon nanotubes are not commercially available in a large quantity due to their high fabrication cost, which limits the application for further improvement in the mechanical properties of RPUFs. Therefore, although the performance of RPUFs could be improved by the above fillers, the environmental and sustainability issues they bring have prompted researchers to turn to eco-friendly fillers [13,14,15]. 

The RPUFs filled with nanoclay minerals are a relatively new composite material. Pal is a natural crystalline hydrated magnesium aluminum silicate with a unique three-dimensional (3D) structure and fibrous morphology. The chemical formula of Pal is Si_8_O_20_Mg(Al)(OH)_2_(H_2_O)_4_·4H_2_O and the chains of Pal are a 2:1 phyllosilicate structure, with each bond connected to the next Si–O–Si bond via a SiO_4_ tetrahedral along with a set of reversals [16]. Pal has been used in a wide variety of commercial applications such as catalysts, rheology agents, decolorizers, and fillers due to its large reservation, low cost, adiabatic properties, and reusability [17,18]. Moreover, Pal has been regarded as an excellent candidate for reinforcement of polymeric materials due to its unique fibrous and nanoscale structural characteristics. However, the hydrophilic nature of Pal results in weak interfacial adhesion to the hydrophobic polymer matrix. Modification of Pal is needed to improve its compatibility and dispersion in the polymer matrix, thus increasing the load transfer efficiency of the system [19,20].

In our previous work, various nanocomposites with improved performance were developed [21,22,23]. In this study, the Pal/RPUFNs were prepared by using the modified Pal as fillers, and the effects of the modified Pal on the properties of RPUFs were assessed, which have rarely been reported in the literature. The mechanical properties, microstructure, and thermal stability of the Pal/RPUFNs were also systematically investigated.

## 2. Experimental Section

### 2.1. Materials

4,4’-Diphenylmethane diisocyanate (MDI) and polyether polyol (including catalysts, silicone oil foam stabilizers, and other additives, with a hydroxyl value of 450 mg of KOH/g) were purchased from Shanghai Guangsheng Thermal Insulation Building Materials Co., Ltd. (Shanghai, China). The silane coupling agent, KH570 (γ-methacryloxypropyltrimethoxysilane) was obtained from Nanjing Chuangshi Chemical Group Co., Ltd. (Nanjing, China). The raw Pal nanofibers originated from Jiangsu Province, China. Glacial acetic acid (AR) was provided by Tianjin Damao Chemical Reagent Group Co., Ltd. (Tianjin, China). Absolute ethanol (AR) was supplied by Tianjin Jiangtian Chemical Technology Group Co., Ltd. (Tianjin, China). Deionized water was produced by the laboratory. All materials were used directly without further purification.

### 2.2. Synthesis of the Modified Pal

The preparation method of modified Pal is briefly described here. First, 10 g of Pal was added into a beaker containing anhydrous ethanol and deionized water with a volume ratio of alcohol to water of 9:1, and stirred at a speed of 800 rpm for 10 min. Next, a certain volume of glacial acetic acid solution was added dropwise to adjust the pH of the suspension to 4 and 0.4 g of silane coupling agent KH570 was added into the solution. Then, the above suspension was transferred to the water bath and stirred for 2 h at 60 °C. After washing with ethanol several times to remove the residual KH570, the suspension was filtrated and dried at 80 °C overnight to obtain the modified Pal. The obtained powder was ground until it could pass through the sieve with 200 meshes prior to use.

### 2.3. Synthesis of the Pal/RPUFNs

The Pal/RPUFNs were prepared by a direct mixing method, in which the polyether polyol and MDI were dried separately at 40 °C for 1 h before use. First, a certain amount of the modified Pal was added to polyether polyol (2 wt%, 4 wt%, 6 wt%, 8 wt%, and 10 wt% of polyether polyol) and mixed at a speed of 1800 rpm in a mechanical stirrer followed by 20 min of ultrasonic treatment. Then, MDI was added in the above polyol mixture with a mass ratio of MDI:polyol = 10:11 and stirred vigorously at 2000 rpm for 10 s. Next, the mixture was immediately poured into a closed stainless-steel mold with a size of 150 × 100 × 100 mm^3^ for molding at room temperature for 30 min to generate foams. The Pal/RPUFN samples were finally obtained after demolding and drying at 60 °C for 12 h. The samples were named respectively as PR0, PR2, PR4, PR6, PR8, and PR10 according to the added amount of the modified Pal.

### 2.4. Characterizations

**Figure 1:**
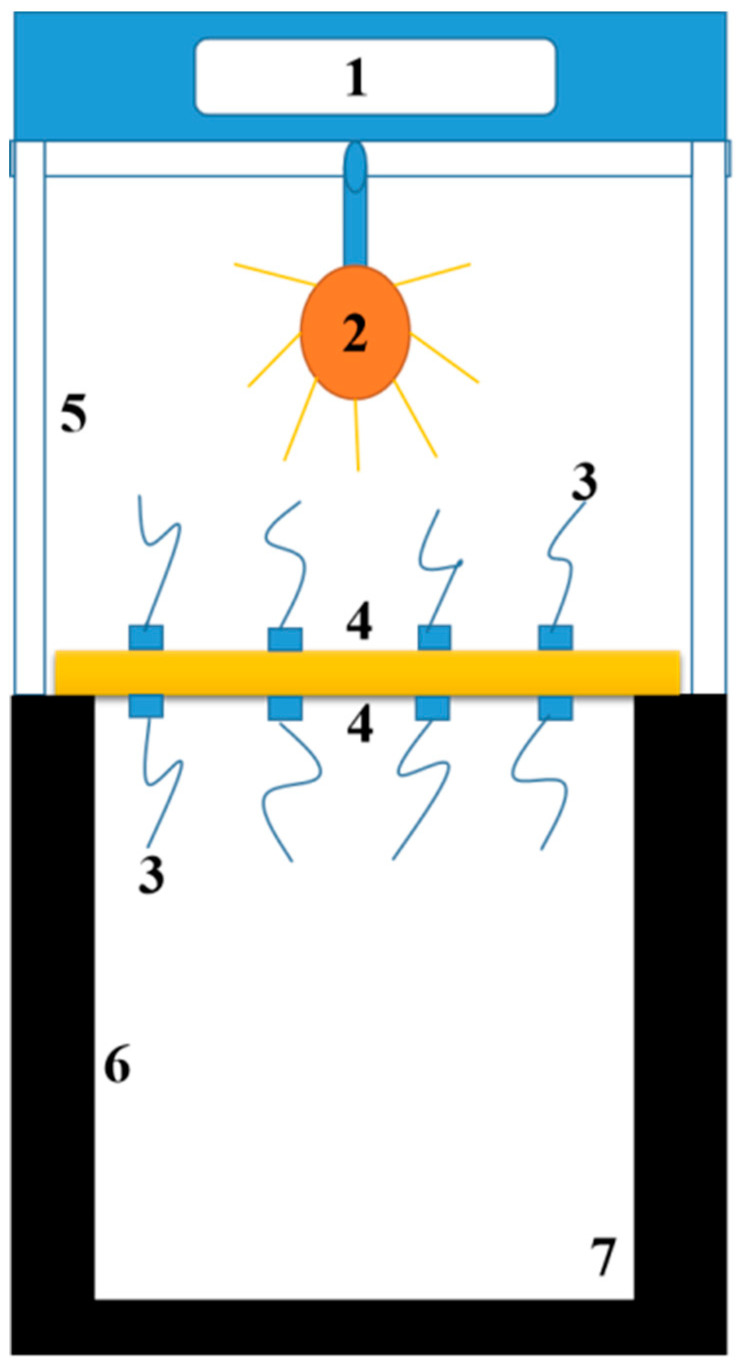
Schematic diagram of the self-made device for estimating thermal insulation: (1) digital display, (2) heating source, (3) thermal resistance, (4) sample, (5) heating chamber, (6) incubator chamber, and (7) insulation layer.

The powder X-ray diffraction (XRD) patterns were recorded by a D8 FOCUS (Bruker, Bremen, Germany) with Cu Kα radiation and collected in 2θ range between 5° and 50°. The apparent density of the RPUF was measured based on GB/T 6343-2009 standard [24]: with the ratio of the sample weight to the sample volume. The sample was cut into several cubes with the dimensions of 40 × 40 × 40 mm^3^ and the mass was measured by an electronic balance with an accuracy of 0.1 mg. The average apparent density of five cut cubes was presented as the final result. The compressive strength of the RPUF was measured based on GB/T 8813-2008 standard [25]. The slope of the linear segment of the stress–strain curve was taken as the modulus of elasticity of the RPUF. Sample was also cut into several cubes with a size of 40 × 40 × 40 mm^3^ prior to the tests. The equipment used for the mechanical property test was a microcomputer-controlled electronic universal testing machine with a model number of CMT6104, maximum test force of 10 kN, a power of 0.4 kW, and a voltage of 220 V, produced by Shenzhen Nss Laboratory Equipment Co., Ltd. (Shenzhen, China). The compression rate was 2 mm/min. The average value of the test data of five samples was used as the final compressive strength, and the error was determined according to the five values contained in each group of data. The morphologies of the samples were observed by scanning electron microscopy (SEM) (Nano SEM450, FEI Co., Ltd, Hillsboro OR, USA) under an accelerating voltage of 2.00 kV after spraying gold. Fourier-transform infrared (FTIR) spectrophotometer (Bruker-TENSOR II) (Bruker Co., Ltd, Billerica, MA, USA) was used to perform Fourier-transform infrared spectrophotometric analysis in the wavenumber range of 4000–400 cm^−1^. The samples were placed in an oven at 60 °C for 24 h before the test. Thermogravimetric analysis (TGA) was performed on an SDT-Q600 simultaneous TG-DTG instrument (TA Instruments Co., Ltd, Newcastle, DE, USA). The samples were heated from room temperature to 800 °C at a speed of 10 °C/min^−1^ under a flow of 60 mL/min of N_2_. The illustration of a self-made device for estimating thermal insulation is shown in Figure 1, where 1 is a digital display instrument; 2 is a heating source; 3 is thermal resistance; 4 is a sample with the size of 300 × 300 × 25 mm^3^ to be tested; 5 is a heating chamber; 6 is an incubator chamber; and 7 is an insulation layer which was filled with thermal insulation materials. The test process is shown as follows: (1) Placed the sample on the location marked with number 4 and closed the upper door tightly; (2) Turned on the infrared light and started the computer automatic counting program; (3) Stopped the program after 1 h, turned off the light, and then took out the sample. Took the average of the temperatures corresponding to the upper and lower surfaces of the sample and calculated the temperature difference between the upper and lower surfaces to evaluate the insulation effect of the sample. The average temperature difference of the three samples was presented as the final result.

## 3. Results and Discussion

**Figure 2:**
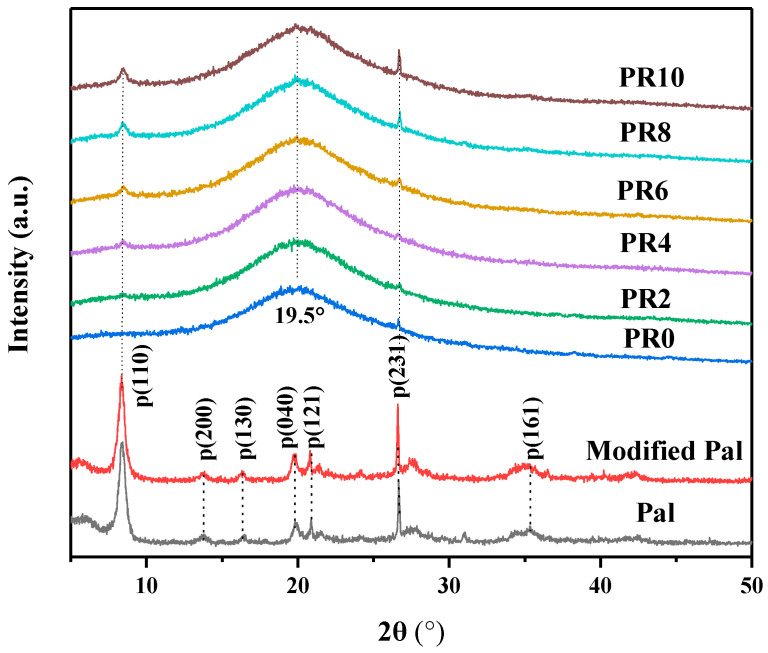
XRD patterns of the raw Pal, modified Pal, and the Pal/RPUFNs with various modified Pal contents.

The crystal structures and phases were analyzed according to XRD patterns. The XRD patterns of the original Pal, modified Pal, RPUF, and Pal/RPUFNs with various modified Pal contents are shown in Figure 2. It can be seen that several diffraction peaks are located at 2θ = 8.4°, 13.9°, 16.4°, 19.8°, 21.4°, 26.5°, and 35.3°, corresponding to the (110), (200), (130), (040), (121), (231), and (161) facets of Pal, respectively. The peaks of the modified Pal remained unchanged, which indicates that the crystal structure of the Pal had not been changed or destroyed after the surface modification. As for all the nanocomposites, a broad diffraction peak is observed at 2θ = 19.5°, which was caused by the short-range order in the arrangement of amorphous polyurethane segments [26]; while for the Pal/RPUFNs no new peak is observed, suggesting that both the Pal and RPUF both retained their original crystal structures. In addition, the peak intensities for Pal (110) and (231) facets gradually increased with the increasing content of the modified Pal.

**Figure 3:**
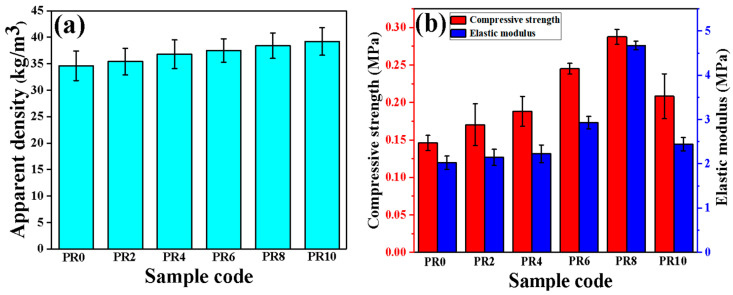
Apparent density (**a**), compressive strength, and elastic modulus (**b**) of the Pal/RPUFNs with various modified Pal contents.

The effects of the filler content on the apparent density of the RPUFs were also investigated based on GB/T 6343-1995 standard. As seen from Figure 3a, the apparent density of the Pal/RPUFNs is increased with the increase of the modified Pal content, which can be attributed to the much higher density of the Pal compared with the pure RPUFs, and the higher viscosity of the mixtures compared with the pure foams [27].

**Figure 4:**
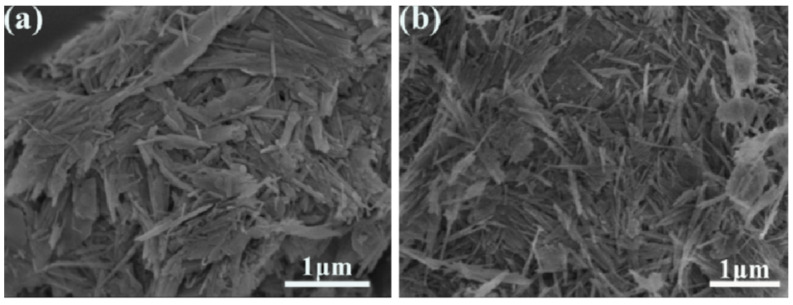
SEM images of the raw Pal (**a**) and modified Pal (**b**).

The mechanical properties of the RPUFs are particularly important in practical applications. They are influenced by many factors, such as cell size, cell wall thickness, density, and test speed [28]. The mechanical properties of the Pal/RPUFNs with various contents of the modified Pal are shown in Figure 3b. The pure RPUFs possess a compressive strength of ca. 0.15 MPa and an elastic modulus of ca. 2.02 MPa. As for the Pal/RPUFNs, both the compressive strength and elastic modulus are increased compared with the pure RPUFs. Notably, the mechanical properties of Pal/RPUFNs exhibit a ladder shape with the increased modified Pal content, and the PR8 reaches the maximum compressive strength (ca. 0.29 MPa) and elastic modulus (ca. 4.67 MPa). According to the SEM analysis shown below (Figure 4), the maximum compressive strength and elastic modulus are attributed to the smaller cell diameter and higher number of cell walls developed in the PR8, which are favorable for its capacity of bearing load. When the modified Pal content reaches 10 wt%, the cell diameter becomes larger and the size distribution also becomes uneven, causing the decreased mechanical properties. The above results indicate that the modified Pal can significantly enhance the mechanical properties of RPUFs, and the optimal addition content of modified Pal is 8 wt%.

The morphologies of the raw Pal and modified Pal were observed through SEM analysis. The original Pal is needle-rod-like aggregates with low dispersion (as shown in Figure 4a) due to its large surface energy, while greatly improved dispersion is observed for the Pal after the modification with KH570 (Figure 4b), and the modified Pal retains its needle-rod-like shape. Combining with FTIR analysis, it is evident that KH570 can not only graft the organic groups on the Pal surface to improve compatibility between Pal and RPUFs matrix, but also promote the dispersion of Pal in the RPUF’s matrix. Thus, the modified Pal is more conducive to be fillers than the original Pal.

**Figure 5:**
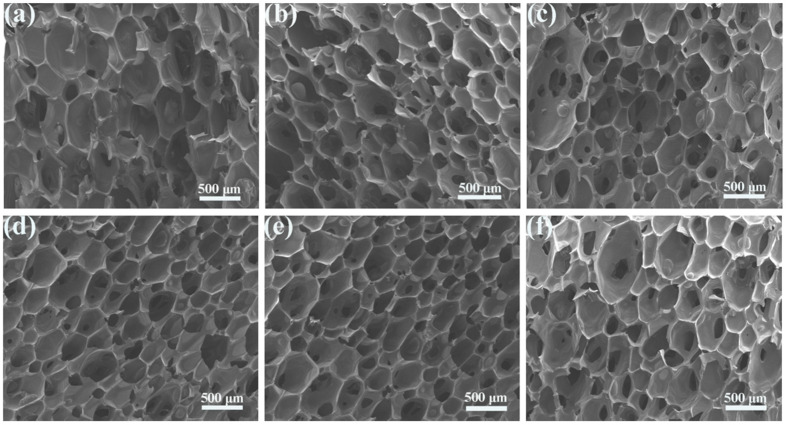
SEM images of the Pal/RPUFNs with various modified Pal contents. ((**a**) PR0; (**b**) PR2; (**c**) PR4; (**d**) PR6; (**e**) PR8; (**f**) PR10).

**Figure 6:**
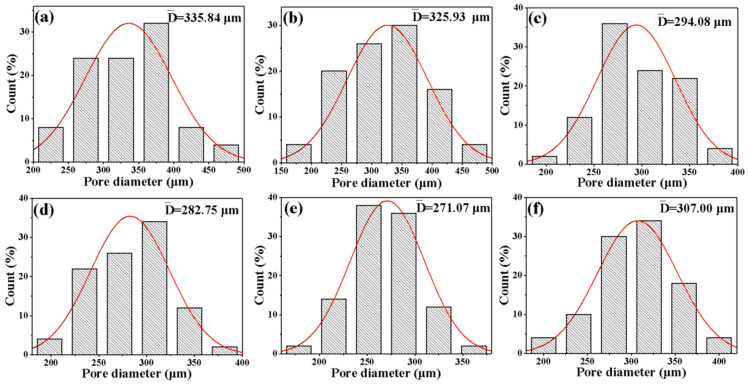
Cell diameter distribution and the fitting curves of the Pal/RPUFNs with various modified Pal contents. ((**a**) PR0; (**b**) PR2; (**c**) PR4; (**d**) PR6; (**e**) PR8; (**f**) PR10).

It is well known that the cell morphology of RPUFs plays an important role in determining their thermal insulation ability and mechanical properties, while the viscosity of raw materials, reaction temperature, and the dispersion of fillers in the foam matrix are critical for the cell structure of RPUFs [29,30]. The cross-sectional images of the Pal/RPUFNs filled with different contents of the modified Pal are shown in Figure 5. In order to quantitatively express the pore diameter of the composites, the related software was used to measure the bubble pore diameter, and the statistical results are shown in Figure 6. It can be seen that both the pure RPUFs and Pal/RPUFNs with various modified Pal contents are composed of closed-cell structure and polyhedral cell walls, and the Pal/RPUFNs with various modified Pal contents exhibit a significantly smaller cell diameter than the pure RPUFs. When the content of the modified Pal is lower than 8 wt%, the average cell diameter becomes smaller with the increased content of the modified Pal. The minimum average cell diameter reaches 271.07 μm for the sample PR8, which is ca. 19.3% smaller than that of the PR0. Moreover, all cells in the PR8 have a cell diameter of less than 400 μm, while the average cell diameter becomes a little larger when the content of the modified Pal is beyond 8 wt%. These phenomena can be explained by the nucleation and growth mechanism [31]. In general, the highly dispersed modified Pal can act as a nucleating agent to transform the nucleation mode from a homogeneous one to a heterogeneous one, which will not only increase the number of nucleation sites, but also greatly reduce the free energy of nucleation. Therefore, the formation of the small cells can be promoted with the support of the modified Pal. Meanwhile, the presence of filler (i.e., modified Pal) increases the reaction rate between the raw materials and limits the growth of cells, which is also beneficial for the formation of smaller-sized cells. However, when the content of the modified Pal is greater than 8 wt%, the viscosity of the foam system increases greatly. This is in contrast to the uniform dispersion of the modified Pal in the RPUFs matrix, resulting in the partial agglomeration and the internal stress or imbalanced growth of foam. Thus, the average cell diameter of the foam increases. In summary, the dispersion of the modified Pal and the chemical reaction between the modified Pal and the RPUFs matrix play an important role in controlling the cellular structure of the foam.

**Figure 7:**
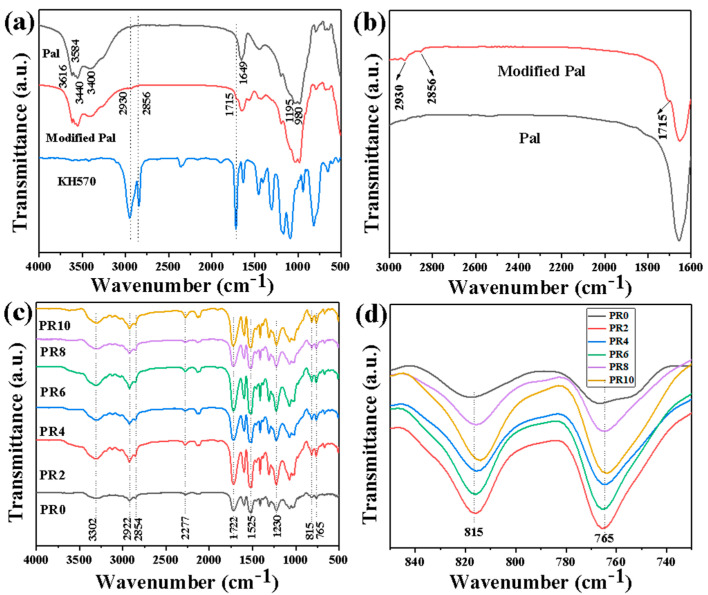
Infrared spectra of the Pal, modified Pal, KH-570 (**a**); the partial amplification view of a (**b**); Pal/RPUFNs with various modified Pal contents (**c**); and the partial amplification view of c (**d**).

The infrared spectra of the Pal and modified Pal are shown in Figure 7a,b. For the original Pal, the stretching vibrations near 3616 cm^−1^, 3584 cm^−1^, and 3550 cm^−1^ are attributed to the hydroxyl [32]. The peaks at 3400 cm^−1^ and 1649 cm^−1^ are attributed to the coordination water and zeolite water, respectively. The tensile vibrations near 980 cm^−1^ and 1195 cm^−1^ are attributed to the Si–O–Si [18]. For KH570, the peaks located at 2930 cm^−1^ and 2856 cm^−1^ correspond to C–H stretching vibrations, and the peak at 1715 cm^−1^ is caused by C=O asymmetric stretching vibration [33,34]. It is worth noting that the peaks corresponding to C–H and C=O of KH570 appear in the modified Pal, indicating the successful surface modification of Pal with KH570. The infrared spectra of Pal/RPUFNs with various modified Pal contents are shown in Figure 7c,d. It is clear that no obvious new peaks appear when the addition content of the modified Pal is changed, indicating that both the modified Pal and RPUFs in the Pal/RPUFNs retained their original structure, and it is consistent with the XRD analysis. The peaks near 3302 cm^−1^, 2922 cm^−1^, 2854 cm^−1^, 2277 cm^−1^, 1722 cm^−1^, 1525 cm^−1^, and 1230 cm^−1^ are attributed to the carbonate N–H group, CH_3_, CH_2_, –N=C=O, C=O, C=C of the aromatic ring, and C–O–C in the RPUFs [31], respectively. Among them, the intensity of the peak located at 2277 cm^−1^ firstly becomes weak and then strong with the increased modified Pal content. This is mainly because of the reaction between the abundant hydroxyl groups from the modified Pal and –N=C=O from the RPUFs at first, while the high addition content of the modified Pal increases the viscosity of the polyether polyol and thus hinders the following reaction between MDI and polyether polyol. Conversely, the CH_2_ swing vibration peak located at 815 cm^−1^ and the Si–O–Si stretching vibration peak located at 765 cm^−1^ firstly become stronger due to the superimposed effect of the addition of a small amount of filler; then, the peak intensity decreases due to the increase in or agglomeration of filler [35]. The above-mentioned results confirm the strong chemical interaction in the interface between the modified Pal and RPUF matrix.

**Figure 8:**
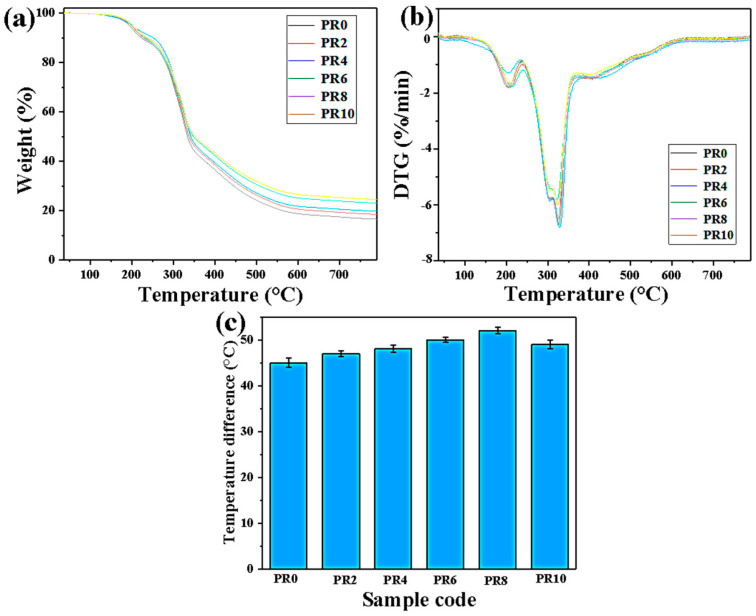
TG (**a**), DTG results (**b**), and the temperature difference between the upper and lower surfaces (**c**) of the Pal/RPUFNs with various modified Pal contents.

To evaluate the thermal stability of the Pal/RPUFNs, TG and DTG curves are presented in Figure 8a and Figure 8b, respectively. Based on the TG and DTG curves, all the nanocomposites exhibit a similar behavior during heating. The thermal decomposition can be divided into three stages: the first stage is the evaporation of water and the volatilization of small molecules such as a foaming agent in the range of 80–200 °C [36,37]; the second stage is the degradation of the hard segment of the polyurethane molecular chain at 200–400 °C, with the weight loss up to 60–70% in this stage [36,38]; and the third stage is from 400 °C to 800 °C, corresponding to the oxidative decomposition of soft isocyanates and aromatic compounds (mainly aromatic rings) in the polyurethane molecular chain. Specifically, the C–H bonds of aromatic rings are broken to form radical fragments containing aromatic ring structures, and then further dehydrogenation and condensation reactions occur between the aromatic rings to eventually form solid carbon-based residues. Furthermore, the temperatures corresponding to 5, 10, and 50% weight loss (named as T_−5%_, T_−10%_, and T_−50%_, respectively) are taken as criteria for evaluating the thermal stability. In addition, the temperature corresponding to the maximum thermal decomposition rate (named as T_max_) is also listed. As shown in Table 1, all the Pal/RPUFNs exhibit higher T_−5%_, T_−10%,_ and T_−50%_ compared with the pure RPUFs. Moreover, PR8 possesses the highest T_−5%_ and T_−50%_ among the Pal/RPUFNs with various modified Pal contents. In addition, all the Pal/RPUFNs show a higher T_max_ value and higher residue rate compared with the pure RPUFs. With the modified Pal content increased, the residue rate continuously rose. The improved thermal stability of the Pal/RPUFNs can be attributed to the following two points: the first point is that a large number of small molecules is generated by polymer thermal decomposition, and the interaction between the small molecules and polymers could accelerate the thermal decomposition of the polymer molecular chain, which is delayed by the match function modified Pal; and the second point is the increased crosslinking density of the Pal/RPUFNs with the help of the modified Pal, which is a remarkable crosslinking agent [39].

The thermal insulation of the Pal/RPUFNs with various modified Pal contents was measured using our self-made instrument, and the temperature difference between the upper and lower surfaces was utilized to evaluate the thermal insulation ability of the nanocomposites. The thermal insulation performance of the pure RPUFs and Pal/RPUFNs is shown in Figure 8c. Similar to the mechanical property, all the Pal/RPUFNs exhibit higher thermal insulation ability than the pure RPUFs, and PR8 also exhibits the highest value. According to previous studies, radiant heat transfer occurs through the cell walls, and the smaller cell diameter can reduce heat transfer through the radiation mechanism. Thus, the cell diameter is crucial for the thermal insulation performance of the RPUFs. Based on the above analysis, the Pal/RPUFNs have a smaller cell diameter and a more uniform cellular structure, which are the main factors for their higher thermal insulation performance, and the PR8 shows the highest performance due to its smallest cell diameter. In addition, the modified Pal itself also has excellent thermal insulation ability. For the Pal/RPUFNs with various modified Pal contents, the modified Pal dispersed in the cell wall can act as a barrier to suppress the heat transfer and reduce the thermal conductivity of the nanocomposites through the conduction mechanism in the solid phase [40]. Overall, the modified Pal filled in the RPUFs can significantly enhance the thermal insulation performance of the RPUFs by decreasing the cell diameter of the RPUFs and suppressing the heat transfer when uniformly dispersed in the cell wall. Finally, the optimal addition content of the modified Pal can be determined as 8 wt%.

## 4. Conclusions

In this work, we firstly successfully synthesized Pal/RPUFNs with different contents of modified Pal. The surface characterization results showed that the modified Pal was uniformly dispersed in the RPUF matrix with strong physical and chemical interactions. Furthermore, the modified Pal had great influence on the cell diameter, mechanical properties, and thermal stability of the Pal/RPUFNs. The elastic modulus and compressive strength of the Pal/RPUFNs were increased by ca. 131% and 97%, respectively, when the modified Pal content was 8 wt%. The thermal stability of the as-prepared modified Pal-reinforced RPUFs was improved, which was reflected in the thermogravimetric analysis of the RPUFs before and after the reinforcement. The synthetic Pal/RPUFNs are expected to exert excellent mechanical properties and thermal stability in practical applications.

## Figures and Tables

**Table 1:** TG and DTG data of the Pal/RPUFs with various modified Pal contents under nitrogen atmosphere.

Sample	T_−5%_ (°C)	T_−10%_ (°C)	T_−50%_ (°C)	T_max_ (°C)	Residual Rate (wt%)
PR0	193	222	333	326	16
PR2	198	228	337	328	18
PR4	200	250	341	330	19
PR6	202	232	347	326	23
PR8	203	237	353	328	24
PR10	200	235	349	327	24

## Data Availability

Data will be available upon request from the corresponding authors.
